# Muscular Systems and Their Influence on Foot Arches and Toes Alignment—Towards the Proper Diagnosis and Treatment of Hallux Valgus

**DOI:** 10.3390/diagnostics12122945

**Published:** 2022-11-25

**Authors:** Jacek Dygut, Monika Piwowar

**Affiliations:** Department of Bioinformatics and Telemedicine, Faculty of Medicine, Jagiellonian University Medical College, Kopernika 7e St., 31-034 Kraków, Poland

**Keywords:** hallux valgus, muscular systems, arches of the foot, tendon stirrup

## Abstract

(1) Background: Static foot deformities, including hallux valgus, are common deformities. The subject under consideration is the role of extrinsic and intrinsic muscles working within muscular systems that shape the arches of the foot and the alignment of the toes. (2) Methods: Based on a literature review, the muscle systems were analyzed. The systems under consideration were as follows: “tendon stirrup” (system I); muscles complementary to the tendon stirrup (system II); “foot lever” (system III); muscles complementary to system III (system IV); “reins of hallux” (system V), a muscular system having distal inserts on the hallux. The shape of the foot arches was analyzed in this context. (3) Results: The correct arch architecture of the foot stabilized mainly by the extrinsic muscle systems determining the function of the intrinsic muscle systems of the foot is described. The proper function of muscular systems shaping the arches of the foot is a prerequisite for the proper function of muscles directly responsible for the alignment of the big toe (hallux) and other foot toes. (4) Conclusion: The action of muscles should be considered in groups (systems) because the action of the group of muscles results in the creation of a new quality of movement. The analysis of individual muscle-pulling forces, especially the moments of force in the weight-bearing foot, may lead to extremely incorrect conclusions. In pathological cases, the restoration of the correct arches of the foot guarantees the recovery of the correct function of the pulling forces of the foot muscles responsible for the physiological alignment of the hallux. This is especially important concerning conservative and surgical treatment of hallux valgus.

## 1. Introduction

There are cases of hallux valgus with normal arches of the foot, but their statistical amount in the population is much smaller than that of cases with the presence of transverse flat and/or plano-valgus foot. Many years of research presented in scientific reports have dealt with the issue of static foot deformities. Causes of static deformities of the foot include overstraining and weakening of the muscles which stabilize the transverse and longitudinal arches of the foot (mainly extrinsic muscles) and stretching of the ligamentous-capsule apparatus under the influence of the gravitational factor or other external forces (e.g., tight footwear or high-heeled shoes) [1,2]. The result is a disturbance of the balance of the muscle-pulling strength at the foot level and, consequently, the onset of foot deformities [3]. Despite the enormous effort, the causes of hallux valgus, plano-valgus, and transverse flat foot are still not fully understood [4,5]. When it comes to the pathomechanisms of hallux valgus described in the literature, most of the research focuses on the hallux valgus itself and the anatomical areas of the big toe. The starting point is the lateral shifting of the big toe as a result of the deforming action of, e.g., footwear. The attention is mainly focused on muscle contractures and disturbing the course of the muscles along the chord with inserts at the level of the phalanges of the big toe [6]. Some researchers state that transverse flat foot and flat valgus foot are secondary to hallux valgus [7].

There are also scientific reports that highlight the complexity of static hallux valgus pathologies, indicating that this is a complex, progressive, multiplanar deformation of the entire foot [7,8]. Other reports also deal with the influence of the arches of the foot upon the course of muscles and the correct alignment of toes [9,10]. Despite accurate observations regarding the complexity of the pathological process of hallux valgus, it is often narrowed down to structures within the foot itself, without taking into account the influence of myofascial structures outside the foot. The analysis of static deformities requires taking into account the extrinsic muscles of the foot. Correct arches of the foot ensure the correct pulling force of the muscles of both the extrinsic and intrinsic muscles of the feet, and this determines the physiological alignment of the toes. Proper foot architecture, in addition to passive stabilizers, is ensured by a balanced isometric contraction of mainly the external muscles of the foot. This, in turn, ensures the alignment of the tendons of the muscles whose distal attachments are on the phalanges of the toes. 

The paper describes the muscle systems, directly and indirectly, responsible for the shape of the foot and the alignment of the toes. Physiological alignment of the big toe is indirectly determined by the proper function of the dynamic systems such as the following:

- System I—“tendon stirrup”—(m. fibularis longus and m. tibial anterior);
- System II—muscles complementary to the tendon stirrup (m. tibial posterior and m. fibularis brevis);
- System III—“foot lever”—(m. fibularis longus, m. tibial posterior, m. tibial anterior, and m. calf triceps);
- System IV—muscles complementary to system III (m. flexor hallucis longus; m. flexor digitorum longus, m. fibularis brevis);
- System V—“reins of hallux”—muscles directly responsible for the proper alignment of the hallux (m. flexor hallucis longus and brevis, m. extensor hallucis brevis, m. adductor, and m. abductor hallucis).

A common feature of muscle systems I-II is that their proximal inserts are outside the foot (thigh, lower leg) while their distal inserts are at the level of the foot and none of these muscles end at the toe phalanges. Their main task is to stabilize the longitudinal and transverse arches of the foot, ensuring correct architecture. In addition, muscle systems I–III provide a physiological course for the foot muscles, which have distal inserts located on the phalanges of the big toe and are directly responsible for the proper alignment of the hallux (system V). System IV, in cooperation with system III, stabilizes the medial longitudinal arch and influences the alignment of the toes, including the hallux. 

## 2. Method

The literature in the area of anthropological, anatomical, and physiological knowledge related to the function of the muscles of human feet were reviewed. Professional literature was from the period time 1904–2020 and was mainly obtained from a PubMed database. Only the most important literature items are cited in the paper. Based on the knowledge and analysis, an assessment of the influence of external and internal muscles on the architecture of the foot and the toe alignment was carried out. The focus was on static foot deformities, mainly hallux valgus.

The implemented methodology was based on the data on the relative strength (%) of individual external and internal muscles of the foot [11] and their arm forces calculated in relation to the axis of the joints on which these muscles act [12]. Based on the value of the force (F) and the length of the force arm (R) for an individual muscle, the moment of force (Mo) produced by the muscle was calculated (Mo = F × R). Due to the fact that the relative force of the muscles given by Silver has the maximum value, and the arm lengths of individual muscles are constant, the interpretation of the moments of force referred to the maximum values. Moments of force generated by individual muscles as well as in antagonistic and synergistic muscle systems were analyzed. The principle was that the moments of force on both sides of the joint rotation axis for individual muscle systems were balanced (F1 × R1 = F2 × R2). In this way, the moments of force produced by direct muscle pulling force were compared. In addition, in the case of a weight-bearing foot, the interpretation includes the moment of gravity (MW = W × r, where W is the weight of the body and r is the distance from the axis of rotation of the joint), which is balanced by the moment of the muscle under eccentric contraction (W × r = F × R). Taking into account the compensatory moment of force of the antagonist muscle being in the concentric contraction being on the same side as the moment of gravity, the formula W × r + F1 × R1 = F2 × R2 was used. By balancing the moment of gravity by the given muscles, the position of the projection of the center of gravity concerning the foot arch was determined (indirect muscle action).

## 3. Results

### 3.1. Dynamic Stabilizing Muscle Systems Responsible for the Foot Arches

#### 3.1.1. System I—Tendon Stirrup—Pair of Muscles: Fibularis Longus and Tibial Anterior

**Figure 1:**
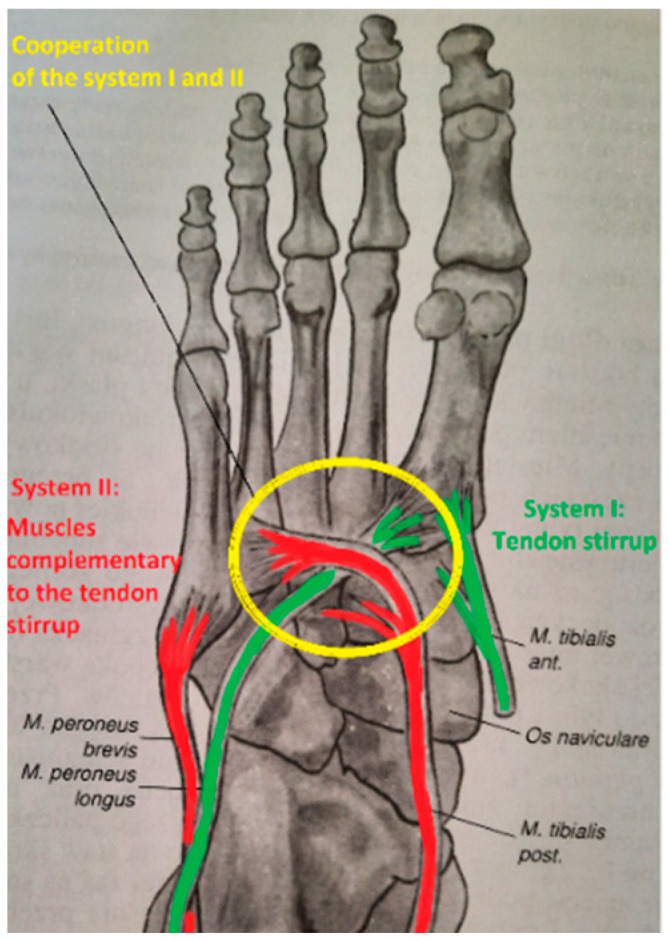
Two systems of the muscles. System I (green)—tendon stirrup consisting of tibial anterior and fibularis longus muscles. System II (red)—muscles complementary to the tendon stirrup consisting of the m. tibial posterior and m. fibularis brevis. Muscles from system I and system II that cooperate are marked with a yellow circle. Own elaboration based on a drawing from [13].

**Figure 2:**
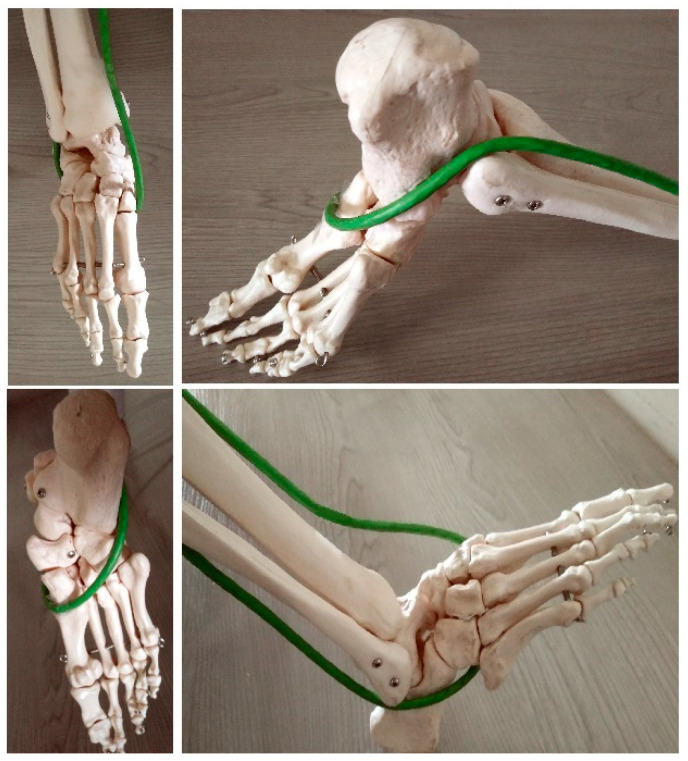
Model of the tendon stirrup (projections of the four basic surfaces of the foot). Two tendons, namely the anterior tibial and fibularis longus muscles, having adjacent inserts on two bones (at the base of metatarsal bone I (plantar side) and medial cuneiform bone) functionally form one system (green loop), constraining the transverse arch and lifting the longitudinal arch of the foot.

The tendon stirrup is a pair of two antagonists, i.e., m. fibularis longus and m. tibial anterior [13,14]. Proximal inserts begin in the lower leg and end at the base of the first metatarsal bone and medial cuneiform bone, on the plantar side. This is the strongest static muscle system involved in shaping the mainly transverse arch. Simultaneous contraction of two antagonists, considering that the peripheral insertions are in the same anatomical area and that their tendons surround the foot on the medial and lateral sides, respectively, brings the bases of the II, III, IV, and V metatarsal bones closer to the base of metatarsal I. The cuboid bone is pushed towards the navicular and lateral cuneiform bone (Figure 1 and Figure 2).

The muscles of the tendon stirrup contract simultaneously, constricting the joints and deepening the foot’s transverse arch at the level of the Lisfranc joint, intertarsal joints, and intermetatarsal joints II-V. One of the tasks of the tendon stirrup is to constrict those joints, preventing them from opening on the plantar side of the foot, and thus flattening the bone-shaped transverse arch in the posterior part (Figure 2). While the flattening of the anterior arch when weight-bearing the foot is a physiological phenomenon, the flattening of the posterior transverse arch should be considered a pathological occurrence.

**Figure 3:**
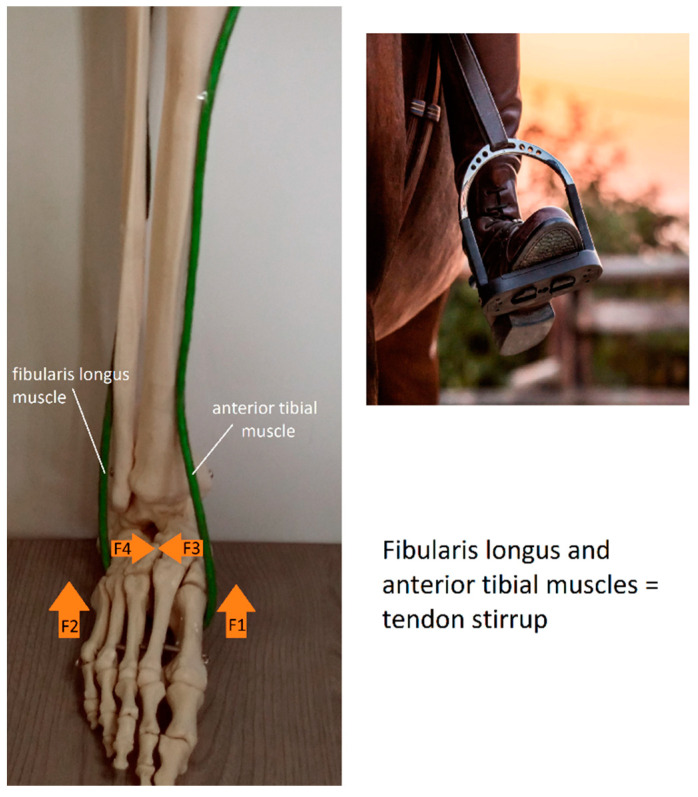
Distribution of pulling forces (F1, F2, F3, F4) presented in the tendon stirrup model (green loop). Vectors F1 and F2 have the same direction; their force values add up to strengthen the lifting force of the longitudinal arch of the foot. Vectors F3 and F4 oppose each other, producing contraction between the lateral and the medial base of the transverse arch, deepening it.

The anatomical transverse arch in the posterior part, shaped by bones and stabilized by the tendon stirrup, transfers through “the bone path” to the heads of the metatarsal bones, thus shaping the anterior transverse arch. The next task of the tendon stirrup is to directly support and even elevate the shaped the posterior transverse arch shaped by the bones. The tendon stirrup results in the narrowing of the foot in the posterior part, simultaneously elevating the medial longitudinal arch and supporting the lateral arch of the foot (Figure 3). In the first phase of contraction, the central part of the foot is narrowed, and in the following phase, the medial longitudinal arch is gradually lifted while supporting the lateral arch [13]. This is because the external muscles that make up the tendon stirrup are the long muscles of the foot and can do more work than the short muscles of the foot of the same strength.

**Figure 4:**
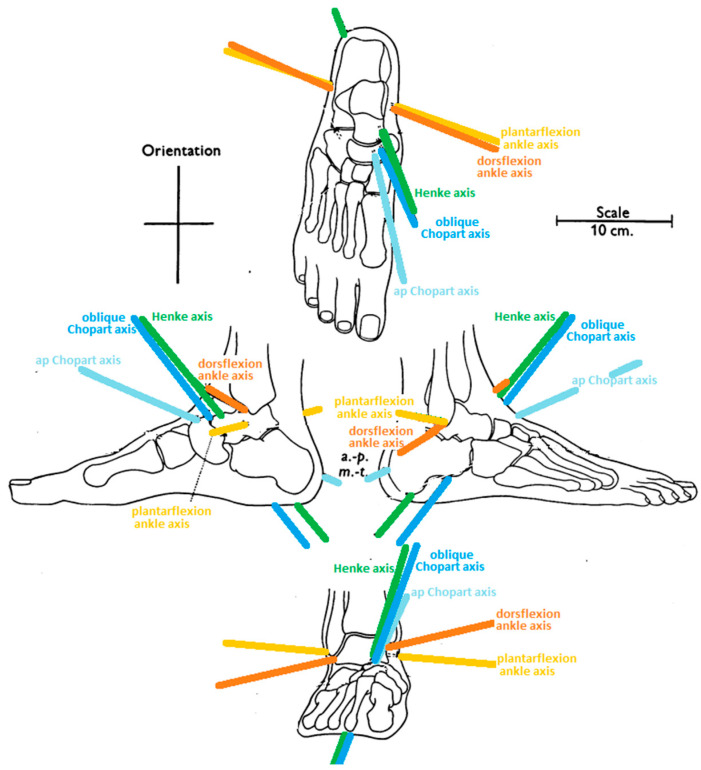
The axes of the joints of the foot: dorsiflexion upper ankle axis; plantar flexion upper ankle axis; Henke axis (lower ankle axis, talocalcaneonavicular axis); oblique Chopart axis (oblique mid-tarsal axis); ap Chopart axis (antero-posterior mid-tarsal axis; longitudinal axis). Own elaboration based on a drawing from [15].

The relative strength (% of the total strength of muscles acting on the foot and ankle) of muscles acting on the foot and ankle is 5.6% in the case of the m. tibial anterior and 5.5% for the m. fibularis longus muscle [8,11]. This would suggest that these muscles are in balance in their action. However, the matter becomes more complicated when considering their moments of force, which depend not only on the magnitude of the force but also on the distance (an arm of force) between the muscle tract and the axis of rotation of the joint. From the level of the base of the first metatarsal bone to the level of the groove of the cuboid bone for the m. fibularis longus, two moments of the force of similar value but with opposite directions are generated. Therefore, the contraction of the m. tibial anterior and m. fibularis longus in this section leads to joints short-circuiting and then supports the transverse arch of the foot. However, it cannot be assumed that if the strength of both muscles is similar, their mutual contraction does not affect foot movement. This is because different moments of force are generated at different articular levels. Based on Hicks’ research, it can be concluded that the maximal moment of force generated by the upper ankle joint by the m. tibial anterior will be twice as large as that generated by the m. fibularis longus (close to the same values of muscle contraction force, but the arm of the force for m. tibial anterior, {−}3 cm, is 2 times larger than the arm of the force for m. fibularis longus, {+}1½, cm in Table 1). The opposite situation occurs concerning the cuneonavicular I joint with the talometatarsal I joint, where the maximal moment of flexing force of the first unit toe (big toe phalanges, first metatarsal bone, medial cuneiform bone) is caused by the m. fibularis longus being twice as high as the m. tibial anterior (the arm of the force is {−}1½ cm for m. tibial anterior and {+}3 cm for m. fibularis longus in Table 1). The muscles of the tendon stirrup do not directly affect the movement in the V unit toe ({---} for tarsometatarsal V joint). The contraction of the m. tibial anterior has no effect on the movement in the lower ankle joint (the arm of the force is 0 in Table 1), while its antagonist, the m. fibularis longus, exerts a significant eversion force (the arm of the force is {−}2 cm in Table 1). The moments of the force of both muscles concerning the Chopart joint are practically canceled out (the arm of the force: {+}1 cm for m. tibial anterior, {−}1½ cm for m. fibularis longus in Table 1). Hicks [12] presented a situation in which the pulling force of the m. tibial anterior directly lies on Henke’s axis (Figure 4).

However, this muscle has a loose tendon sheath, which allows it to shift its pulling force to the medial side from the Henke axis, and the longitudinal axis of the Chopart joint causing it could be a stronger inverter of the Chopart joint, enabling its action as an inverter of the lower ankle joint. This shifting results in a deepening of the height of the medial longitudinal arch in the medial (midfoot) and posterior parts of the foot. When the pulling force of the m. tibial anterior is moved to the lateral side both from Henke’s axis and the longitudinal axis of the Chopart joint, it results in creating an eversion moment that favors plano-valgus setting (Figure 4). Regardless of the position of both axes, the pulling force of this muscle will always extend the first unit toe (inversion of the forefoot) and thus flatten the medial longitudinal arch in the anterior part of the foot.

The balancing of the tendon stirrup is achieved by generating moments of force in the muscles complementary to the stirrup, as well as the m. calf of the triceps. In stabilizing the system, in the zero position of the foot, one should also take into account the moments of force generated by the body weight distributed radially from the basic balance point. By this mechanism of stabilization of the tendon stirrup in a standing position, the entire force of contraction of both muscles is converted into dynamic work (lifting of the arches) and static work (supporting of the arches). Only the m. fibularis longus (the effect of which that is visible outside is insignificant), in the arrangement with the m. tibial anterior, raise the longitudinal arch, and supports it. Not only does it cause the first metatarsal head to take over the greater part of the body mass, but at the same time, the tendency to flex the first metatarsal helps to reduce the stretching of the longitudinal arch and relieve the windlass mechanism [12]. Despite the significant everting force of the hindfoot through the m. fibularis longus, which tends to lower the medial longitudinal arch, the forces deepen the arch in the anterior part (forefoot eversion associated with the flexion of the first unit toe in the cuneonavicular I joint and in the talometatarsal I joint). In some situations, the m. fibularis longus results in the flattening of the forefoot. This is when the moment of force of the m. fibularis longus leading to the flexing of the first unit toe, i.e., the deepening of the foot arch in the anterior part, is countered by the moment of gravity, which is moved forward and outweighs the moment of muscle force. Whether or not the forefoot will be flattened depends on the advantage between the flattening moment of gravitation and the lifting moment due to the flexing of the first unit toe. The isolated action of the m. fibularis longus cannot be considered in static conditions because it must act with its antagonist, i.e., the m. anterior tibial. Otherwise, it would not be able to generate a pull-in force, i.e., a force shortening the rear transverse arch and lifting the medial longitudinal arch of the foot. In an isolated action, the non-weight-bearing foot would seek abduction and an eversion of the foot and resulting in the flattening of the midfoot and rearfoot, with the deepening of the longitudinal arch in the forefoot only in the maximum flexion position. The action of both muscles (at the maximum contraction of both muscles) forming the tendon stirrup in the free foot enables the full pronation movement, i.e., the maximum dorsiflexion in the upper ankle joint with maximum eversion and abduction on the lower ankle and midfoot level.

#### 3.1.2. System II—Complementary Muscles to the Tendon Stirrup: M. Tibial Posterior and M. Fibularis Brevis

**Figure 5:**
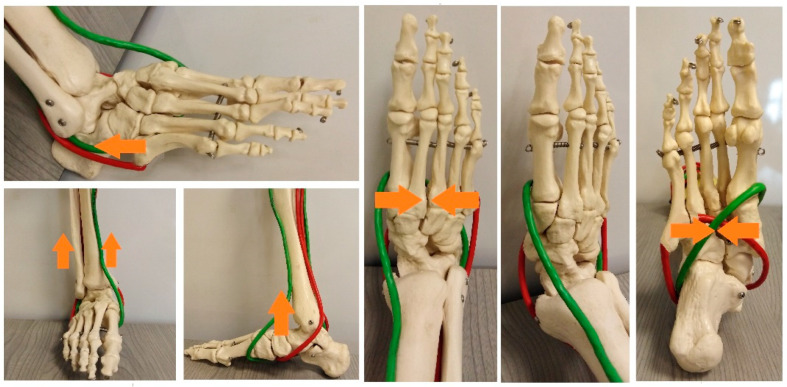
Model of the tendon stirrup (green) (muscle system I) and complementary muscles (red) (muscle system II) in various projections. The two muscle systems, due to their work in pairs, first produce pulling forces that constrict the posterior transverse arch and then support and lift the longitudinal arch. Arrows show the direction of the pulling muscle.

The function of the tendon stirrup is complemented by a pair of antagonists, i.e., the m. tibial posterior and m. fibularis brevis (Figure 1 and Figure 5). The constrictive force of the transverse arch in the posterior part, produced by complementary muscles, is lower than the constrictive force generated by the muscles of the tendon stirrup. The difference is that the complementary muscles to the tendon stirrup do not have attachments located in the same anatomical region, and the distal tendon fibers of the m. tibial posterior reaching the base of metatarsal bones II, III, and IV are thin and weak. Therefore, they do not have such an effective force surrounding the foot as the muscles of the tendon stirrup. On the other hand, the moments of force generated by the complementary muscles to the tendon stirrup have a much greater influence on supporting the longitudinal arch of the foot than the muscles of the tendon stirrup.

Independent action of the m. tibial posterior muscle inverts, adducts, and flexes the foot. When this movement is blocked by the m. fibularis brevis—an evertor (the strongest evertor of the foot) as well as a flexor and abductor—the longitudinal arch of the foot is effectively supported. The m. fibularis brevis acts convergently with the m. fibularis longus; i.e., it crosses the axis of the upper ankle joint from the back, thus acting as a flexor, and laterally from the axis of the lower ankle joint (Henke’s axis), thus acting as an evertor/abductor.

The distances of the muscle pulling line from the axis of rotation of individual joints (i.e., the arms of forces) are similar and, e.g., are {+}2 cm for the m. tibial posterior in relation to the lower ankle joint and {−}2½ cm for the m. fibularis brevis. Similar values of the distance between the pulling lines of these muscles were observed in relation to the Chopart joint ({+}2½ cm for m. tibial posterior and {−}2 cm for m. fibularis brevis) (Table 1). The difference between them is that the muscle pulling line produced by the m. tibial posterior is on the medial side of the axis of rotation of the lower ankle joint and Chopart’s joint (hence the {+} in front of the number), and that produced by the m. fibularis brevis is on the lateral side (hence the {−} in front of the number). However, due to the relative strength of the m. tibial posterior (6.4%) being about 2.5 times greater than that of the m. fibularis brevis (2.6%), the maximum moment of force of the m. tibial posterior is also 2.5 times greater [8,11]. For this reason, the medial longitudinal arch is raised in the non-weight-bearing foot. In addition, it is favored by the much greater flexibility of the medial longitudinal arch than the lateral arch [7]. On the other hand, in the weight-bearing foot, the medial longitudinal arch is strongly supported by the m. tibial posterior. Hicks showed in experimental studies of the weight-bearing foot that the m. tibial posterior muscle cannot actively lift the medial arch, but it is the most important muscle supporting this arch [12].

The excess force is counterbalanced by the remaining extrinsic muscles of the foot and to a lesser degree by the intrinsic muscles of the foot. However, it should be remembered that, in contrast to the constant value arm of the force, the contraction force of the muscle is variable and depends on the number of activated motoric units. By this, the muscle can develop various moments of force depending on the needs (e.g., adjusting the arrangement of the footplate (*lamina pedis*) to the unevenness of the ground).

Muscles complementary to the tendon stirrup cannot actively dynamically raise the medial arch of the foot in a standing position but are the main system supporting the medial arch in a weight-bearing foot.

#### 3.1.3. Cooperation of Systems I and II

The cooperation of selected muscles from systems I and II may also be considered. Regarding the dynamic shaping of the transverse arch of the foot, the interaction of the two antagonists, namely the m. tibial posterior and m. fibularis longus muscles (Figure 1) (Figure 5), whose distal parts are crossing should also be taken into account. Simultaneous contraction of both muscles results in bringing the base of metatarsal bone I (insert of fibularis longus) closer to the bases of metatarsal bones II, III, and IV (inserts of the tibial posterior) and the cuboid bone. The mechanism of this crossing stabilizes the posterior transverse arch and thus assists the tendon stirrup.

#### 3.1.4. System III—“Foot Lever”: Fibularis Longus, Tibial Posterior, Tibial Anterior, and the Calf Triceps

**Figure 6:**
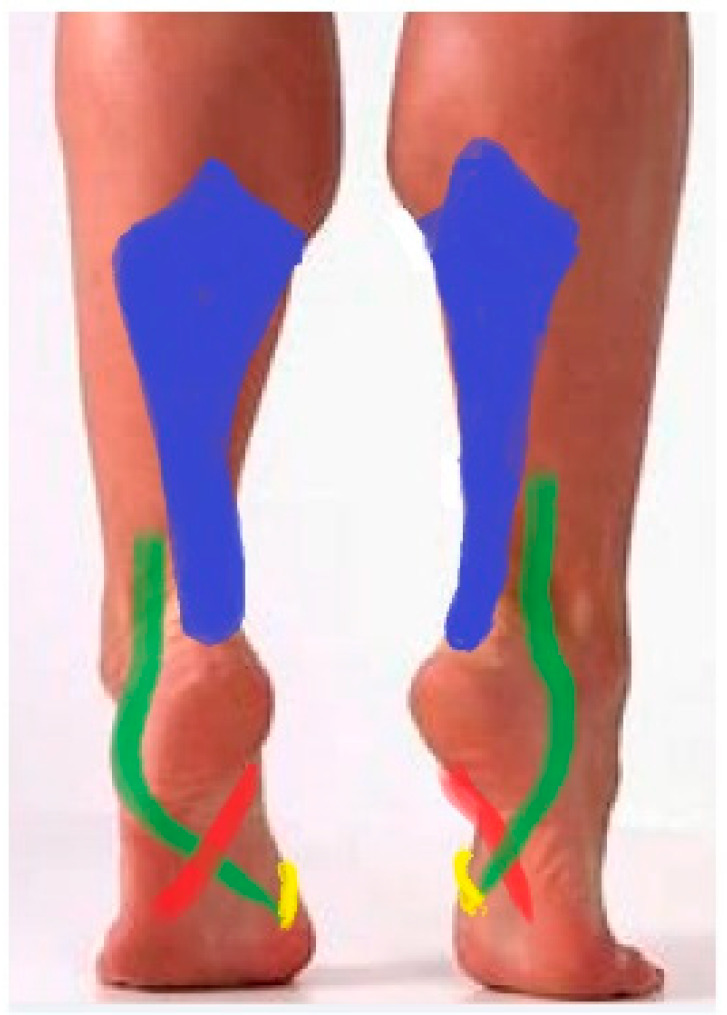
Muscles of system III: m. calf triceps (blue), m. fibularis longus (green), m. tibial posterior (red), m. tibial anterior (yellow). System that lifts, supports, and shapes the medial longitudinal arch as well as stiffing the lever of the foot.

System III consists of the m. fibularis longus, m. tibial posterior, m. tibial anterior, and m. calf triceps muscles (Figure 6), whose task is to lift, support, and shape the medial longitudinal arch and. by stiffening the foot, create a rigid lever capable of pushing off during gait. The shaping of the medial longitudinal arch of the foot is based on the mechanism of the forefoot eversion (m. fibularis longus) and the inversion of the midfoot (m. tibial anterior and m. tibial posterior) as well as the hindfoot (m. calf triceps and m. tibial posterior). It is also based on the flexion mechanism of the Chopart joint.

The m. fibularis longus flexes the first unit toe at the level of the cuneonavicular I joint and talometatarsal I joint, being the main mechanism for lifting the longitudinal arch in the distal part of the foot ({+}3 cm in Table 1). The flexion mechanism of the first toe unit is the eversion mechanism of the forefoot, in which the first toe unit exhibits plantar flexion [16].

The m. tibial posterior is described as the main muscle supporting the medial longitudinal arch. It shapes the arch by exerting flexion force at the level of the Chopart joint (short arm of the moment force) and to a much greater extent by the inversion of the Chopart joint and the lower ankle joint ({+}2½ cm and {+}2 cm, respectively, in Table 1).

The m. calf triceps is the strongest muscle that elevates the medial longitudinal arch. This is due to the influence of this muscle on the lower ankle joint, in which the pulling force of this muscle is located medially from the axis of Henke and backward from the axis of the upper ankle joint (Figure 4). In a static position, this makes the strong adduction and inversion of the calcaneus bone with a simultaneous slight flex (heel supination on the lower ankle level) possible. The supination movement of the calcaneus caused by the Achilles tendon results in the lowering of the articular surface of the calcaneus for the cuboid bone towards the plantar side, and hence the lowering of the lateral longitudinal arch. The other two components of supination, i.e., adduction and inversion, move the cuboid bone towards the medial side and invert it. In the mechanism of the so-called gearbox (“tarsal mechanism”) [5], the navicular bone rigidly connected by the ligaments (*lig. cuboideonaviculare plantare et dorsale*) with the cuboid bone is lifted upwards and towards the lateral side, which significantly deepens the medial longitudinal arch in the midfoot part. The navicular and cuboid bones are almost immobile relative to each other [17].

The above-described mechanism functions not only in the heel-rise and toe-off phases but also in the standing position; however, the standing position is less intensive. This is due to the fact of the distance of the pulling muscles from Henke’s axis. In the static phase, the pulling muscles of the m. tibial posterior and m. calf triceps are located medially from Henke’s axis. In a dynamic situation (gait), the pulling force of these muscles moves away from Henke’s axis more medially, strengthening this mechanism (Figure 4).

The m. calf triceps is the strongest muscle that flexes the foot at the level of the upper ankle joint. Its relative strength is 29.9% for the m. soleus and 13.7% and 5.5% for the m. gastrocnemius medial head and lateral head, respectively [11]. Because the pulling force line of the m. calf triceps slightly deviates medially from Henke’s axis (Figure 4), it is also a moderate invertor of the lower ankle joint (the arm of the force {+}1 cm in Table 1). This muscle, however, acquires a significant inverting force acting in tandem with the m. tibial posterior, which through its contraction shifts the Achilles tendon towards the medial side, increasing its arm of the inverting force.

**Figure 7:**
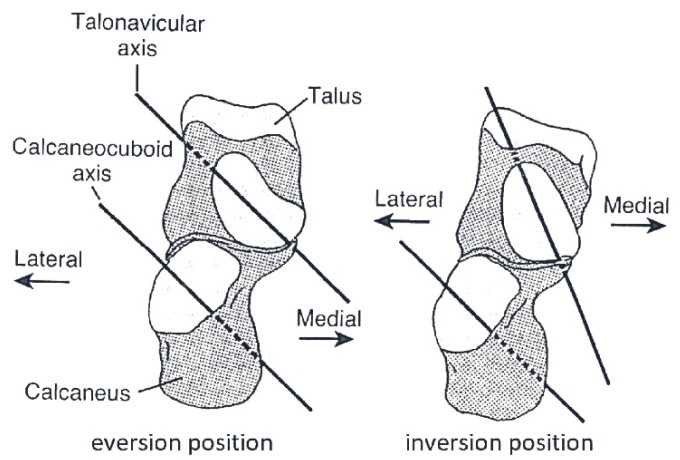
“Close-packed” (inversion) and “loose-packed” (eversion) positions of the midfoot. As the lower ankle joint inverts, the axes of the calcaneocuboid joint and talonavicular joint are convergent, thus locking the Chopart joint and providing rigidity to the midfoot in a “close-packed” position (the inversion position occurs from the standing phase to toe-off, as well as in a static standing position). When the lower ankle joint everts, the axes are parallel, and thus the midfoot is in a “loose-packed” position. Own elaboration based on a drawing from [19].

Physiological inversion of the foot caused by the m. posterior tibial results in a medial displacement of the calcaneus tuberosity along with the Achilles tendon insert (heel inversion) [18]. Thereby, the course of the Achilles tendon is medially positioned along the chord of the pulling force of the m. calf triceps, significantly supporting the inversion of the foot caused by the m. posterior tibial. In this way, the joints forming the medial longitudinal arch of the foot become rigid and the arch deepens. As the lower ankle joint inverts, the axes of the calcaneocuboid joint and talonavicular joint are convergent, thus locking the Chopart joint (transverse tarsal joint) and providing rigidity to the midfoot in a “close-packed” position (Figure 7) [19].

The supination component of both the m. calf triceps and m. tibial posterior in the static position counteracts the body weight, which tries to place the foot in the pronation position [14]. The cooperation of these two muscles is especially emphasized in the heel-rise phase of the gait. The function of the m. tibial posterior is to invert the lower ankle joint and midfoot joint during heel-rise, push-off, and toe-off [8,20]. Loss of this muscle results in acquired pes planus with flattening of the arch, abduction of the forefoot, and eversion of the heel [7,8,21,22]. M. tibial posterior, through its inverting action on the Chopart joint and the lower ankle joint, is the main muscle that initiates the locking of the midtarsal “from behind”, but finally the m. calf triceps causes the midfoot to be blocked. A similar inversion-based mechanism initiates the m. tibial anterior “from the front” (midtarsal locking from the front mechanism).

Under static conditions in a standing position, the tibialis anterior muscle pulling line force is on Henke’s axis and medially from the longitudinal axis of Chopart’s joint (Figure 4). This arrangement favors the inversion of the Chopart joint (the arm of the force is {+}1 cm for the Chopart joint, and the arm of the force is {0} cm in the lower ankle joint, Table 1), and thus it increases the convergent position of the axes of the talonavicular joint and calcaneocuboid joint to each other. This means that the midtarsal locking mechanism from the rear due to inversion action on the lower ankle joint by the m. calf triceps and m. tibial posterior is fused with the mechanism for locking the midtarsal joint (Chopart joint) from the front due to the moment of force generated by the m. tibialis anterior. Shifting the moment of force of the m. tibial posterior towards the medial side of both axes (Henke axis and longitudinal axis of Chopart joint (Figure 4)) shifts and thus increases the moment of force of the m. anterior tibialis, which stiffens the midfoot at the top of the medial arch and tightens the posterior transverse arch. The inverting action of the m. tibial anterior and m. tibial posterior in both the front and behind locking mechanisms of the midfoot is ultimately enhanced by the inverting action of the m. calf triceps.

#### 3.1.5. System IV—Complementary Muscles to System III—M. Flexor Digitorum Longus, M. Flexor Hallucis Longus, M. Fibularis Brevis

The m. flexor digitorum longus is the strongest muscle that flexes the II-V toes. It supports the longitudinal arch. Its relative strength is 1.8% [11]. This muscle crosses the axis of rotation of the upper ankle joint from behind, so it acts as a flexor, and because it runs medially from the axis of rotation of the lower ankle (Henke’s axis), it also acts as an inverter/adductor. The m. flexor digitorum longus acts very similarly to the m. tibial posterior concerning the midfoot and hindfoot, so it inverts the Chopart joint (the arm of the force: {+}2 cm in Table 1) and the lower ankle joint (the arm of the force: {+}1½ cm in Table 1). Thus, it is a muscle that locks the midtarsal “from the back” in the phase of heel-rise and toe-off, with the moment of force generated by it is about 4.4 times lower than that of the m. tibial posterior. The m. flexor digitorum longus has an individual feature that the other external muscles of the foot do not have, i.e., the plantar flexion ability of the IV and V units toes on the level of IV and V tarsometatarsale joints, respectively. The strong flexing action of the V unit toe (the arm of the force: {+}2 cm in Table 1) results in a significant deepening of the lateral longitudinal arch. This function is especially important in the push-off phase of the gait, as it presses the IV and V metatarsal heads to the ground, providing an anterolateral support point. The m. flexor digitorum longus under the weight-bearing condition shifts the body weight ahead of the rotation axis of the upper ankle joint (the arm of the force: {+}1½ cm in Table 1) and laterally to the axis of Henke (the arm of the force: {+}1½ cm in Table 1) and the longitudinal axis of Chopart’s joint (the arm of the force: {+}2 cm in Table 1), creating a balance point above the lateral arch in the anterior region. No effects on the longitudinal arch were observed in the experimental studies. This is because the inversion of the midfoot and rearfoot and shifting of the weight-bearing forward and laterally result in a lowering of the lateral longitudinal arch. At the same time, the V unit toe is flexed and the lateral longitudinal arch is deepened. In total, these effects cancel each other out.

The m. flexor hallucis longus (extrinsic muscle) is one of the three deep muscles of the posterior compartment of the lower leg that attach to the plantar surface of the distal phalanx of the big toe. The primary action of the m. flexor hallucis longus in the non-weight-bearing foot is flexion of the big toe on the level of the I metatarsophalangeal and interphalangeal joints (the relative strength of this muscle is 3.6% [11]). In a weight-bearing foot, the contraction force of the m. hallucis longus results in the flexion of the first unit toe (the arm of the force: {+}1½ cm in Table 1) and the pressing of the big toe’s pad against the ground. It has been proved in experimental studies that the common action of the m. flexor hallucis longus and m. fibularis brevis gives a similar effect to the action of the m. fibularis longus [12]. The m. flexor hallucis longus, on its running on the plantar side of the foot, crosses from the dorsal side to the tendon of the m. flexor digitorum longus (*chiasma plantare*), extending a tendon strand to it (usually directed to the II and III toes, and sometimes also to the IV toe). As a result, the m. flexor hallucis longus flexes not only the big toe but also the other toes. It plays an important role when lifting on the toes. The m. flexor hallucis longus acts on the upper ankle joint and performs plantar flexion of the foot (the arm of the force: {+}2 ½ cm in Table 1); its action leads to adduction and inversion of the lower ankle joint (the arm of the force: {+}1 cm in Table 1). It also causes inversion and adduction and flexion at the level of Chopart’s joint (the arm of the force: {+}1½ in Table 1). Many publications erroneously conclude that the tendon of the m. hallucis longus, running in the sulcus tendinis musculi hallucis longi processus posterior tali and under the sustententaculum tali, lifts the anterior part of this bone, making it steeper and deepening the posterior part of the medial longitudinal arch. In fact, there is a supination of the calcaneus, i.e., flexion, adduction, and inversion. The articular surface of the calcaneus, for the cuboid bone, lowers and moves towards the medial side so that the navicular bone rises upwards and laterally. Finally, the medial longitudinal arch rises with the simultaneous lowering of the lateral longitudinal arch [16]. The tendon of the m. flexor hallucis longus running between the tuberculum mediale and laterale of the posterior processor of the talus produces a resultant force “pushing” the talus to the dorsal and distal sides of the foot, preventing its posterior subluxation. It is not true that running of the tendon under the sustentaculum tali produces a resultant force that lifts (extension) the anterior part of the calcaneus and the caput of the talus. The caput of the talus is elevated indirectly as a result of the elevation of the navicular bone in the mechanism of the so-called gearbox [5] and directly by inverse positioning of the sustentaculum tali (upward and lateral displacement). The m. flexor hallucis longus strengthens and elevates the medial longitudinal foot arch and protects against plano-valgus.

#### 3.1.6. System V—“Reins of Hallux”—Muscles Directly Responsible for the Position of the Hallux, i.e., the Flexors and Extensors of the Hallux and the Abductor of the Hallux

The correct positioning of the hallux is directly influenced by the m. flexor and m. extensor hallucis longus (extrinsic muscles), together with the m. flexor and m. extensor hallucis brevis and a pair of antagonists, i.e., the m. abductor and m. adductor hallucis (intrinsic muscles). A characteristic feature of these muscles is the location of the distal inserts within the big toe, providing its proper alignment. Correct functioning of muscles is possible when the foot architecture, including the longitudinal and transverse arches, is correct.

(a) The flexor and extensor muscles of the hallux (“drive reins”)

The flexor and extensor muscles of the hallux are mainly active during the forward gait and thus are described as “drive reins”.

The m. flexor hallucis brevis is a short muscle (intrinsic muscle with a relative strength of 1.5% [11]) located on the plantar side of the foot. It is attached between the plantar side of the I–III cuneiform and navicular bones, plantar calcaneocuboid ligament, both sesamoid bones, and the proximal phalanx of the big toe (hallux). In the free foot, it flexes the big toe, while under weight-bearing conditions, it generates a direct force under the first toe unit (flexing of the first toe unit) of the foot, which supports the lifting of the medial longitudinal arch of the foot.

The m. flexor hallucis longus is described in system IV.

The m. extensor hallucis longus (extrinsic muscle with a relative strength of 1.2%) ends at the dorsal surface of the distal phalanx of the big toe. The muscle is a strong extensor of the hallux and the foot at the level of the upper ankle joint. Depending on the position of the tendon relative to the axis of the lower ankle joint (Henke’s axis), it may weakly evert (the arm of the force: {−}1 cm in Table 1) or invert the rearfoot, thus affecting the flattening or deepening of the longitudinal arch of the foot in the posterior part (Figure 4). This is due to the opportunistic nature of this muscle, the distal insertion of which may move from the medial to the lateral side or lie on the axis of Henke. Due to its direct course over the Chopart joint (when the pulling force is on the axis of Henke), it does not generate torque (the arm of the force: 0 cm in Table 1). Therefore, it does not affect the shape of the central part (midfoot) of the medial longitudinal arch of the foot. Depending on the situation, when the pulling force is off the Henke axis, a slight inversion/eversion movement is possible. The course of the m. extensor hallucis longus directly over the first toe unit results in the flattening of the medial longitudinal arch in the anterior part. It is a consequence of the elevation (extension) of the first toe unit (the arm of the force: {−}1½ cm in Table 1), which is equal to the inversion of the forefoot. The muscle action described above relates to a situation where the hallux is against the ground and the muscle is in an eccentric contraction with the line of gravity moved towards the heel (flexion position of the foot). In a situation when the big toe is set dorsally (extension), the windlass mechanism is activated, resulting in pressing the head of the first metatarsal bone against the ground (deepening the longitudinal arch in the anterior part) [15].

The m. extensor hallucis brevis (intrinsic muscle) is a short muscle located in the dorsum of the foot, attaching between the calcaneus and proximal phalanx of the hallux. Working in synergy with the m. extensor hallucis longus, the main function of this muscle is to assist in the extension of the big toe at the metatarsophalangeal joint I.

The m. flexor and m. extensor muscles of the big toe are responsible for the flexion or extension of the hallux in the sagittal plane at the level of the metatarsophalangeal I and interphalangeal joints. These muscles play a special role during gait. The m. flexor hallucis longus and brevis are active in the push-off phase, while the m. extensor hallucis longus and brevis are active in the swing phase. It should be noted that the arms of the force of both muscles in relation to the axis of rotation of the upper, lower, and Chopart ankle joint as well as the axis of the first toe unit are almost identical, but located on both sides of the above-mentioned axis. Besides this, the difference between these muscles is their relative strength, which is 3.6% for the m. flexor hallucis longus, 1.2% for the m. extensor hallucis longus, 1.6% for the m. hallucis brevis, and 0.6% for the m. extensor hallucis brevis. This shows that flexor muscles are more than 3 times stronger than extensor muscles because their role is to perform static work related to the formation of the medial longitudinal arch of the foot and dynamic work related to the rebound of the foot in the toe-off phase (“forward drive”).

(b) M. adductor hallucis and m. abductor hallucis (“direction reins”)

The m. adductor hallucis and m. abductor hallucis are muscles responsible for the direction of the hallux; hence, they are described as “direction reins”.

The m. abductor hallucis begins on the medial side of the calcaneus, the plantar fascia, the tuberosity of the navicular bone, and the plantar surface of the cuneiform medial bone and ends at the medial sesamoid bone and the base of the proximal phalanx on the medial side. It is the strongest short muscle of the foot that shapes the medial prominence of the sole (relative strength: 2.7% [11]). The muscle strengthens the medial, most convex part of the foot arch. In addition, it flexes the big toe and moves it away (abducts) from toe II.

The m. adductor hallucis is a biceps muscle with relative strength for the oblique head equal to 2.3% and the transverse head equal to 0.4% [11]. A thick and long oblique head (*caput obliqum*) begins at the bases of metatarsal bones II-IV, the lateral cuneiform bone, the cuboid bone, and the long plantar ligament. The fibers produce a roundish muscle belly that fuses with the lateral muscle belly of the flexor hallucis brevis muscle. The flat and thin transverse head (*caput transversum*) begins at the capsules of metatarsophalangeal joints II-V and the deep transverse metatarsal ligaments. The fibers run in the medial direction. Both heads end at the lateral sesamoid bone and the base of the proximal phalanx of the big toe. This muscle replaces the action of the plantar interosseous muscle: it adducts the big toe to toe II and flexes it at metatarsophalangeal joint I.

The balance between m. abductor hallucis and m. adductor hallucis (intrinsic muscles) ensures the alignment of the hallux in the horizontal (plantar) plane and supports the transverse and longitudinal arch of the foot. This occurs mainly in the case of a stabilized I metatarsophalangeal joint and blocked plantar flexion of the big toe (e.g., when standing and in the phase of gait support). It should be emphasized that the total contribution of the m. adductor hallucis and m. abductor hallucis to the stabilization of the foot arch is less than that of the tendon stirrup muscles or the “foot lever” muscles (extrinsic muscles of the foot).

The main action of the m. adductor hallucis is antagonistic to the m. abductor hallucis, enabling proper alignment of the hallux. It supports the action of the tendon stirrup, the main stabilizer of the foot’s transverse arch. The transverse head of the m. adductor hallucis deepens the transverse arch, thus narrowing the forefoot, and the oblique head deepens the longitudinal arch, shortening the foot. However, the overall contribution of the adductor and abductor hallucis to the stabilization of the arches of the foot is lower than that of the tendon stirrup muscles and other extrinsic muscles of the foot.

“Direction reins” are relatively strong muscles. However, because they are shorter than the external muscles of the foot, they can perform mainly static work and to a much lesser extent dynamic work. Hence, their main function is to keep the big toe in the correct position and support the foot arches.

## 4. Discussion and Conclusions

Proper foot architecture ensures optimal muscle work, with the physiological positioning of the toes. The proper arched shape of the foot guarantees the correct course of pulling muscles, the inserts of which end at the phalanges of the hallux. When analyzing the pathomechanism of static foot deformities, muscles should be considered in groups since the action of pairs or larger groups of muscles—often antagonists—results in complex phenomena, which cannot be deduced from the movement of individual muscles. Some researchers do indeed analyze the function of foot muscles in pairs and larger groups, but even these systems do not take into account interactions that give rise to new qualities in terms of movement [23]. The function of each is analyzed in isolation, leading to wrong conclusions.

The dominant view in many anatomy textbooks is that the m. fibularis longus and brevis flex the foot in the plantar direction and evert and abduct the foot. Both fibular muscles, along with the m. fibularis tertius, are described as flat foot muscles because the action on the lower ankle joint everts and abducts the foot. If it were so, this positioning of the foot would favor the “sliding” of the talus from the calcaneus. Hicks’ experimental research clearly shows that the m. fibularis longus is not a flat foot muscle. On the contrary, it is responsible for supporting the medial longitudinal arch, and also its lifting [12] due to the large everting force of the forefoot at the level of the I cuneonavicular joint (as a result of I toe unit flexing). The duplicated error results from the analysis of the muscle in its single action, disregarding its antagonist. Description of the non-weight-bearing foot is incorrectly transferred to the conditions of the weight-bearing foot, which is affected by the moments of the force of other muscles and the moment of gravity.

A similar situation applies to the extensor muscles of the foot, including the m. tibial anterior, which is the strongest extensor of the foot. In the non-weight-bearing foot, it can be considered that the muscle that extends the upper ankle joint can cause the flattening of the medial longitudinal arch because its direct pulling force is above the first toe unit. In the weight-bearing foot, when the projection of the center of gravity is shifted beyond the axis of the upper ankle joint, eccentric contraction of the m. tibial anterior balances the moment of gravity. Thanks to this, the weight is shifted towards the heel, and the flattening force is significantly reduced, which indicates that under weight-bearing conditions, the m. tibial anterior may paradoxically deepen the medial longitudinal arch of the foot. It may also contribute to the medial longitudinal arch deepening in the midfoot due to its inverting effect on the Chopart joint [12], and when its pulling force lies medially to Henke’s axis, it is also an invertor of the lower ankle joint (Figure 4).

The m. tibial anterior (due to the wide and loose tendon sheath) can shift from the medial to the lateral position to the Henke axis, thus changing itself from the invertor to the evertor. The opportunistic nature of the work of this muscle with the eversion and inversion of the footplate (*lamina pedis*) is especially required when walking on uneven ground. In some anatomical books, the imprecise statement that it acts synergistically with the m. tibial posterior and prevents the formation of flat feet is incorrectly duplicated. This is true of only the inversion action of this muscle on the Chopart joint and the lower ankle joint. One forgets about its strong inversion effect on the forefoot which is conducive to flat feet. The distal segment of the tendon of this muscle runs from the medial side to the plantar surface of the base of the first metatarsal bone and the medial cuneiform bone, so the action of this muscle always ultimately results in an inversion of the forefoot (extension of the I unit toe).

Often, the assessment of the hallux valgus deformity is limited to examining the deviation of the big toe along the first metatarsal or interphalangeal joint and examining the course of muscles with inserts on toe phalanges (system V). In our opinion, the analysis of static foot deformities should be extended by the assessment of the distribution of pulling forces and force moments of extrinsic muscles, and moments of gravity for Henke’s axis and the longitudinal Chopart axis (inversion/eversion; abduction/adduction) as well as the upper ankle joint axis and oblique Chopart axis (flexion/extension) (Figure 4) [12]. The cumulative moments of the forces of the pulling muscles and moments of gravity must be balanced on both sides of the axis of joint rotation. This analysis shows whether the foot arches have the correct shape. Based on such an examination, we can determine whether hallux valgus occurs as an independent deformity, with correct arches of the foot, or is accompanied by a transverse flat and/or plano-valgus foot. Many publications emphasize that mainly the m. abductor hallucis strengthens the medial longitudinal arch of the foot and the transverse head of the adductor hallucis muscle strengthens the transverse arch of the foot [24]. The influence of intrinsic muscles on shaping the arches of the foot is disproportionately lower than that of extrinsic muscles. This follows from Fick’s principle (1911) [8,25], which says that muscle strength is proportional to its physiological cross-section. It also depends on the arm of force, i.e., the shortest distance between the line of muscle action and the joint axis which influences the moment of the force [14]. Moreover, the longer the muscle, the longer the contraction path is and the greater the work it does.

The basis for correction of static deformities, e.g., hallux valgus, transverse flat foot, and plano-valgus foot, is the balancing of the action of muscle systems I-V responsible for the correct shape of the transverse and longitudinal arches of the foot and the proper alignment of toes.

## Figures and Tables

**Table 1:** The value of the arm of force is expressed in centimeters. It is measured from the axis of rotation of a given joint to the pulling of a given muscle (the shortest distance). The symbols {+} and {−} indicate the position of the tendon in relation to the joint axis. The upper ankle joint: {+} behind, {−} in front; the lower ankle joint (Henke axis): {+} medially, {−} laterally; Chopart joint (longitudinal axis): {+} medially, {−} laterally. Zero {0} means that the pulling of muscle runs parallel to the joint’s axis of rotation. Lack of data {---} means that the pulling muscle does not cross the axis of the joint and therefore does not affect the joint. Table based on Hicks’ data [12].

Muscle	Upper Ankle Joint	Lower Ankle Joint	Chopart Joint	Cuneonavicular I Joint with Talometatarsal I Joint (First Unit Toe Joint *)	Tarsometatarsal V Joint (Fifth Unit Toe Joint **)
Tibial anterior	{−}3	0	{+}1	{−}1 ½	---
Fibularis longus	{+}1½	{−}2	{−}1½	{+}3	---
Tibial posterior	{+}1	{+}2	{+}2½	---	---
Fibularis brevis	{+}1½	{−}2½	{−}2	---	0
Calf triceps	{+}4	{+}1	---	---	---
Flexor hallucis longus	{+}2½	{+}1	{+}1½	{+}1½	---
Extensor hallucis longus	{−}3	{−}1	0	{−}1½	---

* The first unit toe (big toe phalanges, first metatarsal bone, medial cuneiform bone); ** the fifth unit toe (digiti minimi phalanges, fifth metatarsal bone).

## Data Availability

Not applicable.
